# Supplementary material for: Optimizing rice-fish co-culture: Investigating the impact of rice spacing density on biochemical profiles and production of genetically modified tilapia (Oreochromis spp.) and Cyprinus carpio
Source: PLoS One. 2023 Dec 28;18(12):e0295996. doi: 10.1371/journal.pone.0295996 (PMC10754446; doi:10.1371/journal.pone.0295996)
Supplement: S1 File — (DOCX) [file pone.0295996.s001.docx]

**Plankton’s diversity**

By using plankton net phytoplankton and zooplankton were sampled and their diversity was determined fortnightly [1]. Water sample was collected in the three different corner of paddy field for the identification of plankton. In order to collect Zooplankton samples, 20 liters of mixed water were filtered through a 112-cm plankton net and stored in formaldehyde at 4%. A 1 L mixture of mixed water was withdrawn and an iodine solution was added to collect phytoplankton samples. A laboratory analysis was performed in order to identify the species and determine the biomass volume of phytoplankton and zooplankton. Microscopes (Olympus CX31) and stereoscopes (Olympus SZX10, Japan) were used and with the help of available keys and manual, plankton grouping were identified [2, 3].

**Results**

**Plankton Community**

In rice fish co-culture, plankton communities were measured fortnightly. From phytoplankton, five groups and sixteen genera were observed throughout the experiment (Table 1). A number of individual cyanobacteria (aphanizomenon, oscillatoria, spirulina, gloeotridia, microcystis, nostoc and anabaena), diatoms (synedra, cylindrotheca, navicula and cyclotella), motile algae (spirogyra, pediastrum and chlorella) and green algae (chlymadomonas, trachelomonas) were significantly lower in RHD (low rice spacing density) as compared to other treatments. The numbers of genera were higher in RMD (medium rice spacing density).

In zooplankton, five groups and fourteen genera were observed throughout the experiment (Table 2). The Number of individual Euglena, Ameoba, Paramecium, Apicomplexa, Bosmiina, Moina microra, Daphnia carinata, Cyclops scutifer, Mesocyclopshyalinus, Cyprissubglobosa, Brachionuscalcifiorus, Brachionuscaudatus, Asplanchna, Keratellatropica showed the higher value in RMD (medium rice spacing density) as compared to other treatments groups.

**Table 1.** Phytoplankton community among different treatments groups

| **Group** | **Genera (cell/L)** | **RHD** | **RMD** | **RLD** | **P-value** |
| --- | --- | --- | --- | --- | --- |
| **Cyanobacteria** | Aphanizomenon | 290.00^c^±10.00 | 567.50^a^±4.50 | 375.50^b^±7.50 | 0.0003 |
|  | Oscillatoria | 228.00^c^±12.00 | 411.50^a^±2.50 | 360.50^b^±12.50 | 0.0022 |
|  | Spirulina | 1320.00^a^±33.0 | 1394.50^a^±8.50 | 1386.50^a^±11.50 | 0.14720 |
|  | Gloeotridia | 1503.00^a^±10.50 | 1528.00^a^±7.00 | 1496.50^a^±2.50 | 0.1119 |
|  | Microcystis | 917.50^b^±3.50 | 978.50^a^±7.50 | 827.50^c^±8.50 | 0.0013 |
|  | Nostoc | 264.50^c^±1.50 | 411.50^a^±7.50 | 377.00^b^±5.00 | 0.0006 |
|  | Anabaena | 503.50^c^±8.50 | 690.50^a^±6.50 | 637.50^b^±8.50 | 0.0010 |
| **Diatoms** | Synedra | 465.50^c^±5.50 | 562.50^a^±3.50 | 491.50^c^±1.50 | 0.0008 |
|  | Cylindrotheca | 734.50^c^±5.0 | 869.50^b^±0.50 | 1001.50^a^±9.50 | 0.0002 |
|  | Navicula | 745.30^c^±5.0 | 936.50^a^±10.50 | 877.50^b^±1.50 | 0.0005 |
|  | Cyclotella | 869.00^b^±13.00 | 1021.50^a^±6.50 | 814.50^c^±5.0 | 0.0011 |
| **Green Algae** | Spirogyra | 1755.50^c^±0.50 | 1967.00^a^±7.00 | 1865.50^b^±5.50 | 0.0002 |
|  | Pediastrum | 254.00^b^±8.00 | 390.00^a^±3.00 | 366.50^a^±8.50 | 0.0016 |
|  | Chlorella | 2550.00^b^±67.00 | 2763.00^a^±3.00 | 2662.50^ab^±0.50 | 0.0672 |
| **Motile Algae** | Chlymadomonas | 212.50^c^±9.50 | 370.00^a^±8.00 | 260.00^b^±7.00 | 0.0019 |
|  | Trachelomonas | 261.50^b^±10.50 | 348.00^a^±4.00 | 328.50^a^±10.50 | 0.0127 |

*Means with different superscripts in rows differ significantly at P ≤ 0.05;* ***RHD****= Rice high density (RHD),* ***RMD****= Rice medium density (RMD),* ***RLD****= Rice low density (RLD)*

**Table 2.** Zooplankton community among different treatments groups

| **Group** | **Genera (cell/L)** | **RHD** | **RMD** | **RLD** | **P-value** |
| --- | --- | --- | --- | --- | --- |
| **Protozoan** | Euglena | 123.00^a^±4.00 | 148.00^a^±9.00 | 138.00^a^±4.00 | 0.1349 |
|  | Ameoba | 188.50^b^±12.50 | 227.00^a^±3.00 | 227.00^a^±5.00 | 0.0649 |
|  | Paramecium | 108.00^a^±8.00 | 142.00^a^±5.00 | 123.50^a^±10.50 | 0.1294 |
|  | Apicomplexa | 273.50^a^±10.50 | 298.50^a^±10.50 | 298.50^a^±10.50 | 0.2944 |
| **Cladocera** | Bosmina | 587.00^a^±5.00 | 617.50^a^±3.50 | 604.00^a^±13.00 | 0.1695 |
|  | Moina | 723.50^c^±1.50 | 778.50^b^±6.50 | 813.00^a^±11.00 | 0.0077 |
|  | Daphnia carinata | 308.00^a^±5.00 | 329.00^a^±17.00 | 328.00^a^±13.00 | 0.5030 |
| **Copepods** | Cyclops scutifer | 760.00^b^±12.00 | 826.00^a^±4.00 | 792.50^ab^±13.50 | 0.0500 |
|  | Mesocyclopshyalinus | 252.00^c^±1.00 | 344.00^a^±8.00 | 314.50^b^±4.50 | 0.0026 |
| **Ostracods** | Cyprissubglobosa | 256.50^a^±10.50 | 312.00^a^±11.00 | 286.50^a^±18.50 | 0.1410 |
| **Rotifers** | Brachionuscalcifiorus | 2397.00^c^±12.00 | 2728.00^a^±20.00 | 2561.00^b^±9.00 | 0.0012 |
|  | Brachionuscaudatus | 1097.50^b^±16.50 | 1195.00^a^±17.00 | 1123.50^b^±12.50 | 0.0433 |
|  | Asplanchna | 1270.00^b^±12.00 | 1345.00^a^±5.00 | 1264.00^b^±21.00 | 0.0470 |
|  | Keratellatropica | 262.50^b^±9.50 | 333.50^a^±4.50 | 300.00^ab^±15.00 | 0.0402 |

*Means with different superscripts in rows differ significantly at P ≤ 0.05;* ***RHD****= Rice high density (RHD),* ***RMD****= Rice medium density (RMD),* ***RLD****= Rice low density (RLD)*

**References**

1. Apha A. Wef. Standard methods for the examination of water and wastewater. 2005;21:258-9.
2. Das D, Pathak A, Pal S. Diversity of phytoplankton in some domestic wastewater-fed urban fish pond ecosystems of the Chota Nagpur Plateau in Bankura, India. Applied Water Science. 2018;8(3):1-13.
3. John DM, Whitton BA, Brook AJ, editors. The freshwater algal flora of the British Isles: An identification guide to freshwater and terrestrial algae. Cambridge University Press; 2002.
