# Supplementary material for: Optimizing rice-fish co-culture: Investigating the impact of rice spacing density on biochemical profiles and production of genetically modified tilapia (Oreochromis spp.) and Cyprinus carpio
Source: PLoS One. 2023 Dec 28;18(12):e0295996. doi: 10.1371/journal.pone.0295996 (PMC10754446; doi:10.1371/journal.pone.0295996)
Supplement: Attachment — Submitted filename: Paper review PLOS.doc [file pone.0295996.s002.doc]

**MANUSCRIPT REVIEW SHEET**

**Journal;** PLOS ONE

**Ref No.:** PONE-D-22-31911

**General comments:**

The present research work has no such significant importance and advancement in this field. There is no such novelty in the present study, since this types of study has already been performed.

**Specific comments:**

**Title:** Title should be more clear. Please rrewrite title

**Abstract: Rewrite it clarifying the following comments**

1. The abstract is not self-explanatory.
2. It should be more specific and cclear
3. Where is the results of rice?
4. Novelty statement?

**Introduction:**

1. It is very good and informative.
2. Please insert some relevant current references from other authors if available.
3. “one word, from the perspective of fish and rice, the optimum rice spacing density and optimum fish species for the culture of rice and fish have remained silent.” Is it right ? There are several studies.
4. Need more current references.
5. Rice spacing, density .. is not proper

**Materials and methods**

It is not well structured. Need to separate the rice, fish parameters, water soil parameters

Experimental design should contain fish stocking

*It is ok.*

**Results**

It is not well structured. Presents the results on the basis of important results. Rice and fish production parameters should come first.

Need to rewrite it properly.

Only table, need some graphical presentation.

**Discussion**

I think author should deal with the result-wise (parameter-wise) outcome of the present study supporting previous and current referential study. Need more specific discussion without general statements as mentioned in the starting of the discussion. Need more analytical discussion on the basis of results obtained from study

Insert a clear concluding sentence with your significant supporting evidence obtained.

**Table:** Ok. Check caption properly.

**References:** Not checked. Please check the references.

It is not acceptable at this stage and needed major revision.
