# Supplementary material for: Optimizing rice-fish co-culture: Investigating the impact of rice spacing density on biochemical profiles and production of genetically modified tilapia (Oreochromis spp.) and Cyprinus carpio
Source: PLoS One. 2023 Dec 28;18(12):e0295996. doi: 10.1371/journal.pone.0295996 (PMC10754446; doi:10.1371/journal.pone.0295996)
Supplement: Attachment — Submitted filename: Response to Reviewers.docx [file pone.0295996.s003.docx]

**Response Letter**

The,

Editors,

PLOS ONE

**Subject: SUBMISSION OF REVISED PAPER**

Dear Editor,

Thank you for your email Dated: 22/Dec/2022 enclosing the reviewer’s comments. We appreciate the time and effort that you and the reviewers have dedicated to providing your valuable feedback on my manuscript. We are grateful to the reviewers for their insightful comments on my paper. We have carefully reviewed the comments and have revised the manuscript accordingly. Our responses are given in a point-by-point manner below. We hope the revised version is now suitable for publication and look forward to hearing from you in due course.

You’re sincerely,

**Muhammad Inayat**

**ACADEMIC EDITOR**

I have critically gone through the manuscript and the Ms reviewed by two very efficient reviewers working in the field of capture and culture fishery. Please go through those comments and reconsider to make title more appealing and directly addressing objectives.   Both the reviewers have similar views and I also consider required revision required along the same line.

The manuscript has neither page number nor line number, so I find difficulty in commenting objectively.

**Response:** Thank you for your valuable feedback on our manuscript. We appreciate your feedback and we are grateful for the opportunity to improve our manuscript. All suggested changes by editor and reviewers have been made accordingly. Page number and Line number has been added.

The Ms. has eight tables and three figures, as zooplankton and phytoplankton community are not main goal of the study, I would suggest to shift these tables to supplementary tables (Figure 4 and 5).

**Response:** Agreed. Tables 4 & 5 of Zoo and Phytoplankton have been shifted to Supplementary data.

However, zooplankton like cyclopoid copepods are abundantly found in race paddy field, many species are predators of fish larvae. Do they affect fish production in rice paddy field. This issue may be brought in discussion with references and author's zooplankton data.

**Response:** As author clearly mentioned in section materials and methods, Line No. 118-120, the average weight of stock fish was 50±3.0 g that is much bigger than larvae and couldn’t edible by zooplankton like cyclopoid and copepods.
For Lab, Study and Registered Report Protocols: These article types are not expected to include results but may include pilot data.

**Additional Editor Comments:**
**1.** The manuscript has neither page number nor line number, so I find difficulty in commenting objectively.

**Response:** Agreed. Page number and Line number has been added.

**2.** The Ms. has eight tables and three figures, as zooplankton and phytoplankton community are not main goal of the study, I would suggest to shift these tables to supplementary tables (Figure 4 and 5). However, zooplankton like cyclopoid copepods are abundantly found in race paddy field, many species are predators of fish larvae. Do they affect fish production in rice paddy field? This issue may be brought in discussion with references and author's zooplankton data.

**Response:** As author clearly mentioned in section materials and methods, Line No. 118-120, the average weight of stock fish was 50±3.0 g that is much bigger than larvae and couldn’t edible by zooplankton like cyclopoid and copepods.

**3.** Tables 7 and 8 can be nicely presented as graph as simple vertical bars, X axis: parameters and Y axis: Values (pH values may be given on secondary Y axis or omitted

**Response:** Tables 7 and 8 has been converted into graph and pH values mentioned along Y-axis.

**4.** Proper proof reading has not been done carefully, e.g., Legend of table 1 is given below that table where as legend of table 2, 3 are given above tables. Similarly Figure 2 is inserted within the text but other Figures are given at the end of text, after reference section.

**Response:** Agreed. Revised manuscript proof read twice and all clerical mistakes have been removed.
**5.** The statement appendix of tables as "Superscripts on different means within row differ significantly at P ≤ 0.05; RHD= Rice high 8 density (RHD), RMD= Rice medium density (RMD), RLD= Rice low density (RLD), is not clearly communicate the message what (i) if two means are superscripted by same alphabets and (ii) if two means are superscripted by different alphabets??

**Response:** The appendix of table is revised as Means with different superscripts in rows differ significantly
**6.** Figure 3: What is purpose of line drawing of values, which are not continuous and different points represent different parameters.

**Response:** The graph is now converted into separately presented parameters graphs.
**7.** All sections of the manuscript need restructuring and rewriting. Language is verbose.
A completely rewritten manuscript focusing on objectives of the study may be considered for another round of assessment.

**Response:** The manuscript has been rewritten and revised accordingly.

**Review Comments to the Author**
Please use the space provided to explain your answers to the questions above. You may also include additional comments for the author, including concerns about dual publication, research ethics, or publication ethics. (Please upload your review as an attachment if it exceeds 20,000 characters).

**Reviewer #1: Journal; PLOS ONE
Ref No.: PONE-D-22-31911**
General comments:
The present research work has no such significant importance and advancement in this field. There is no such novelty in the present study, since this type of study has already been performed.

**Response:** The study of the effects of rice spacing density on Tilapia and Carp in the specific context of Pakistan's climate region is an important and relevant topic in the field of rice-fish co-culture. While there may have been previous studies that have investigated this topic in other regions, the specific conditions of the climate and environment in Pakistan can lead to variations in the results. This research is a novel and pioneering study not only in the specific context of Pakistan. the present research work provides a significant contribution to the field of rice-fish co-culture by investigating the effects of rice spacing density on Tilapia and Carp in Pakistan's climate region, which is the first study of its kind in this area and worldwide. It provides new data and insights that can be useful for practical applications, and contributes to the overall understanding and advancement of this topic in this specific region and other regions with similar climates.
Specific comments:
**1.** Title: Title should be clearer. Please rewrite title

**Response:** Title has been changed and precise.
**2.** Abstract: Rewrite it clarifying the following comments
1. The abstract is not self-explanatory.

**3. Response:** Abstract has been improved with clarity of study concept.
2. It should be more specific and clearer

**Response:** Abstract has been revised with specification of study.
4. Where are the results of rice?

**Response:** Comparative results of rice growth and yield has been incorporated in revised abstract.
**5.** Novelty statement?

**Response:** Rice-fish culture is a novel and sustainable aquaculture system that optimize the co-culture of rice with different fish species in Pakistan are most beneficial for the aquaculture industry and enhance agriculture GDP as well.
**6.** Introduction:
1. It is very good and informative.
**7.** Please insert some relevant current references from other authors if available.

**Response:** Relevant and latest reference has been included in introduction.
**8.** “One word, from the perspective of fish and rice, the optimum rice spacing density and optimum fish species for the culture of rice and fish have remained silent.” Is it right? There are several studies.

**Response:** Agreed. Above line has been removed from introduction. Despite the several studies that have investigated the optimal rice spacing density and fish species for rice-fish co-culture, the specific conditions may vary in different regions. The research in this specific climate region of Pakistan is not limited, which makes this study novel and valuable not only for Pakistan but also for other regions with similar climates. This study aims to provide valuable insights and data on the effects of rice spacing density on Tilapia and Carp in Pakistan's climate region which can be useful for practical applications in rice-fish co-culture management in this area and other regions with similar climates.

**9.** Need more current references.

**Response:** As mentioned above, Relevant and latest reference has been included.

**10.** Rice spacing, density.is not proper.

**Response:** As several reference cited in introduction with same term that depicts rice spacing density is proper.

Materials and methods
**11.** It is not well structured. Need to separate the rice, fish parameters, water soil parameters
Experimental design should contain fish stocking. It is ok.

**Response:** Rice, fish, soil and water parameters has been separately incorporated in revised manuscript and fish stocking also added in experimental design.
Results
**12.** It is not well structured. Presents the results on the basis of important results. Rice and fish production parameters should come first. Need to rewrite it properly.

**Response:** Results of rice yield and fish rearing are presenting before the results of soil and water.

**13.** Only table, need some graphical presentation.

**Response:** Table No. 7 & 8 are in graphical form now.
Discussion
**14.** I think author should deal with the result-wise (parameter-wise) outcome of the present study supporting previous and current referential study. Need more specific discussion without general statements as mentioned in the starting of the discussion. Need more analytical discussion on the basis of results obtained from study.

**Response:**  Importance and relevance of present results has been well compared with latest literature review in revised manuscript.
**15.** Insert a clear concluding sentence with your significant supporting evidence obtained.

**Response:**  On the basis of revised discussion, concluding statement has been rewritten now.
**16.** Table: Ok. Check caption properly.
References: Not checked. Please check the references.

**Response:**  All caption of tables, figures and reference has been cross checked.

**Reviewer #2:**

Comments- The manuscript requires rigorous re-editing and proof reading, to ensure clarity. The research work is very common, and using advanced tools to support the data obtained would greatly benefit the research area, and add novelty.
**1.** The title should include the key species used for the experiment.
**Response:** The title of the article has been revised.

**2.** The abstract should provide proper insight about the research, with more clarity.
GMT, RMD, RLD, RHD, are mentioned several times, before the first mention of their unabbreviated form. Maintain uniformity in the use scientific name, or common name.

**Response:** The introduction is informative, and could be re-written to make it better.

**3.** Add more recent references for the general statements. There are very few recent references used in the manuscript.

**Response:** Relevant and latest reference has been included in introduction

Materials and methods
**4.** Is "description of the field" appropriate enough for the specifications of the study area? How is the climate, elevation, and temperature relevant to the research conducted? Numerous grammatical errors are present, and the section could be restructured.

**Response:**
The climate condition of the study area has been added. The grammatical errors are checked.

**5**. Fig 2, the symbols used after A, B, and C, do not match the ones in the figure.

**Response:** The figure has been revised.

**6.** Where were the fishes procured from? Were they acclimatized prior to use in the experiment? If yes, then in what conditions. If no, why not? What was the age of the fishes used in the experiment.

**Response:** The procurement details of fish has been mentioned and acclimatized method also added.

**7.** How were the feeding and growth conditions accessed, to increase the quantity of the feed?

**Response:** For the accessing of growth and feed requirement of fish, regular netting on weekly basis was done. Feed quantity was increased with size and weight of fish.

**8.** Is "evaluation of meat analysis" the best fit?
**Response:** Yes, the meat analysis is best fit.

**9.** How were the zooplankton identified?
**Response:** Identification protocol of zooplankton has been added.

**10.** The materials and methods should be described clearly enough for the work to be reproducible.
**Response:** Yes, Methodology is much clearly described.

Results
**11.** Re-write it more clearly. Some of the scientific names mentioned are italicized, without capitalizing the first word. Some are written with no space between the generic name and specific name.

**Response:** The results of the manuscript has been revised; the scientific names are re-written accordingly.

**12.** The most important results of the study are related to the rice and fish, that should be mentioned first.
**Response:** The results of the rice and fish has been mentioned first.
Discussion
**13.** The discussion has too many general statements, better suited for introduction. Add more scientific discussion and interpretation, relevant to the results obtained.

**Response:** In discussion, more interpretation with relevant results has been revised.

**14.** Alanine, Glycine and such amino acids can be mentioned in their full name, before using Ala, Gly etc.

**Response:** Amino acids has been mentioned with full name before abbreviation**.**

**15.** Re-write, with rigorous proof -reading, to remove elementary level grammatical errors.
**Response:** Revised manuscript has been proof-read and thoroughly checked by grammatically.
**16.** Check PLOS one guidelines are formatting for references and change accordingly.
**Response:** Entire manuscript is revised on Plos one formatting accordingly.
Table
**17.** Use "Species" instead of "Specie"
**Response:** The word specie has been replaced with species.

**18.** Italicize the scientific names.
**Response:** Scientific name has been italicized.

**19.** What are "Ostracodans"?
"Protozoan", "Cladocera", "Copepods", "Rotifers" maintain uniformity.
**Response:** Ostracodon word has been replace with ostracods that are belong to class crustaceans also known as seed shrimp.

**20.** Is "Bosmiina" the correct word?
**Response:** Agreed. Typological error the word Bosmiina has been replaced with Bosmina.

**21**. Why are there line numbers from table 4?
**Response:** Mistakenly line number was added in table 4 that is removed.

**22.** What is the "f" in Line 60

**Response:** The word F is removed now.
**23.** Add line numbers to the entire manuscript, instead of the Table?

**Response:**

**24.** The line number has been added.
