# Supplementary material for: Optimizing rice-fish co-culture: Investigating the impact of rice spacing density on biochemical profiles and production of genetically modified tilapia (Oreochromis spp.) and Cyprinus carpio
Source: PLoS One. 2023 Dec 28;18(12):e0295996. doi: 10.1371/journal.pone.0295996 (PMC10754446; doi:10.1371/journal.pone.0295996)
Supplement: Attachment — Submitted filename: Point by point response to reviewers comments.docx [file pone.0295996.s004.docx]

**RESPONSE LETTER**

The,

Editors,

PLOS ONE

**Subject: SUBMISSION OF REVISED PAPER**

Dear Editor,

Thank you for your email Dated: 7/June/2023, considering my appeal and enclosing the reviewer’s comments. We appreciate the time and effort that you and the reviewers have dedicated to providing your valuable feedback on my manuscript. We are grateful to the reviewers for their insightful comments on my paper. We have carefully reviewed the comments and have revised the manuscript accordingly. Our responses are given in a point-by-point manner below. We hope the revised version is now suitable for publication and look forward to hearing from you in due course.

You’re sincerely,

**Muhammad Inayat**

**[POINT-BY-POINT RESPONSE TO REVIEWER'S COMMENTS](https://acp.copernicus.org/preprints/acp-2020-237/acp-2020-237-AR2.pdf)**

Reviewer comments to the author
**Reviewer #2: 1**. The authors have stated in their response that the research work is the first study of its kind, worldwide, while there are several studies accessing the impact rice spacing densities and fish species in such systems.

**Response:** Yes, authors genuinely meant to suggest that their research is the first of its kind in all aspects, including the variables examined and the methodologies utilized in **Pakistan**. Upon considering your feedback, we recognize that there have been previous studies that have assessed the impact of rice spacing densities and fish species in similar systems. These studies have contributed valuable knowledge to the field. We apologize for any confusion caused by the authors' original statement. However, it is important to note that while previous studies have explored similar variables and methodologies, our research in Pakistan aims to expand upon and complement the existing body of literature. Our study incorporates unique aspects, such as the specific geographic context, local agricultural practices, and potential regional variations.

**2.** The abstract omits the bio-chemical aspect of this research work, and is not self-explanatory.

**Response:** The explanation of bio-chemical profile of fish species has been added in revised abstract.

**3. L99-L102.** How is the climate, elevation, and temperature relevant to the research conducted? An explanation is essential why the above factors may influence the research results.

**Response:** The climate, elevation, and temperature in Pattoki, Punjab, are relevant to research as they impact various aspects of the study. The subtropical monsoon climate influences agriculture, and environmental factors. The maximum elevation affects atmospheric conditions and ecological dynamics. The temperature range influences biological processes, human health, and climate change-related research. Considering these factors is essential for accurate data collection, interpretation, and generalizability of research findings

**4. L163,** the protocol for evaluation of amino acid profile should be mentioned in detail, with the mention of any specific standardization carried out, if any.

**Response:** The revised method of amino acid evaluation has been added in manuscript.
**5.** How were the zooplankton identified, after detecting them under the microscope?

**Response:** Under the microscope, zooplankton is identified by examining their morphological characteristics such as body shape, size, appendages, and coloration. Taxonomic keys and reference materials are used to compare these features with known species.

**6.** The manuscript would benefit from re-structuring the statements, and rigorous proof reading.

**Response:**  We appreciate your valuable input. We have restructured the statements for improved organization and conducted rigorous proofreading to enhance clarity. These revisions have significantly improved the manuscript's coherence and readability.
