# Supplementary material for: Optimizing rice-fish co-culture: Investigating the impact of rice spacing density on biochemical profiles and production of genetically modified tilapia (Oreochromis spp.) and Cyprinus carpio
Source: PLoS One. 2023 Dec 28;18(12):e0295996. doi: 10.1371/journal.pone.0295996 (PMC10754446; doi:10.1371/journal.pone.0295996)
Supplement: Attachment — Submitted filename: Point by point response.docx [file pone.0295996.s005.docx]

**RESPONSE LETTER**

The,

Editors,

PLOS ONE

**Subject: SUBMISSION OF REVISED PAPER**

Dear Editor,

Thank you for your email Dated: 7/Aug/2023, enclosing the reviewer’s comments. We appreciate the time and effort that you and the reviewers have dedicated to providing your valuable feedback on my manuscript. We are grateful to the reviewers for their insightful comments on my paper. We have carefully reviewed the comments and have revised the manuscript accordingly. Our responses are given in a point-by-point manner below. We hope the revised version is now suitable for publication and look forward to hearing from you in due course.

You’re sincerely,

**Muhammad Inayat**

**POINT-BY-POINT RESPONSE TO REVIEWER'S COMMENTS**

**Editor comments**
The following additional aspects need to be taken while re-submitting a revised version:
**1.** Delete all p values from the Abstract. It is sufficient to say that significant differences exist among treatments.

**Response:** The P-value has been deleted from the Abstract.

2. In the title please provide the taxonomical names of fish species and the authority of the taxon. This ensures proper identification of the taxa

**Response:** The title has been revised with the addition of taxonomical names of fish species.

**3.** Last sentence of the abstract change as “The study provides data to understand…

**Response:** The last sentence of the abstract has been changed to “The study provides data to understand”

**4.** Lines 55-56: Many parasites from the field reach the ducks, fish, etc. Mention necessary limitations in the conclusions about this

**Response:** The limitation related to integrated farming has been added in conclusion.

**5.** The pond length for cultivation of rice is shallow but for culturing fish species, higher depth is needed. Make necessary justification

**Response:** Given the requirements of the fish species being cultured, a pond depth of 5-6 feet was accurately chosen. This depth was thoughtfully configured alongside the adjacent rice culture channel to provide an ecologically suitable and conducive environment for the rearing of fish species.

**6.** It is necessary to use post hoc tests following anova. In statistical analysis some anomalous trends have been depicted. For example, Table 1 GMT weight gain in Tilapia and carp the differences shown as significant due to different letters of superscript. This requires confirmation because standard deviation is very large. In the same table, data were presented in real units and in percentage or per day. This is a considerable duplication of information. First column is sufficient.

**Response:** The tables have been changed in the revised manuscript.

**7.** This applies to other tables too.

**Response:** The table has been changed.

**8.** I have to fully checked all the tables and figures. But it is important to avoid presenting the same data in figures and tables.

**Response:** The data has been changed into updated tables.

**9.** Provide results of multiple comparisons for the bars of figs. 3-5 (with alphabets).

**Response:** The figure is now changed into tables.

**10.** I failed to understand why the discrete bars are connected by a dotted line. This gives the impression that continuous data were collected. This is used for variables like temperature.

**Response:** The figure is now converted into new tables.

**11.** Do not start a sentence with a number (line 351).

**Response:** The sentence has been re-write in revised manuscript.

**12.** The ms has 71 references. Weed out excessive refs and restrict to 50 citations. Fig. 1 shows the world map for Pakistan is too basic. Remove this blue square fig.

**Response:** We have made efforts to reduce the number of references, and it now stands at 56. We added some new references in response to reviewer requests.

The Fig. 1 has been revised

**Reviewer #1: Comments:**
1. The title of MS is still not clear.
**Response:** The MS title has been revised.

**2.** The write-up of MS is still not standard.
For example: The write has been revised.
Fat % = Fat weight (g)/weight if sample ×100

**Response:** The formula is that total fat extraction in fish samples is divided by the total weight of fish and then multiplied by 100

Fat Content (%) = (Weight of Fat in the Fish / Total Weight of the Fish) x 100

**3.** Before the harvesting of rice, rice stem characteristics were measured (Fig 3). This sentence can be of M & M part.

**Response:** The above sentence has been added to the revised manuscript

**4.** Crude protein % = Volume ×0.875/sample weight

**Response:** The crude protein is calculated by the Kjeldahl method, the revised formula is added in the manuscript as follows,

**Crude Protein (in %)** = (N x 6.25) / W

Where:

N: Nitrogen content determined from the sample.

6.25: Conversion factor, assuming that proteins contain approximately 16% nitrogen.

W: The weight of the sample in grams.

**5.** Need references for the methods used in study for Yield parameter of rice

**Response:** The reference has been added to the rice yield parameter method.

**6.** Under different rice spacing densities, least square mean ± standard errors of crude protein
and fat of the muscles of the fish are presented in

**Response:** The above sentence has been revised.

**7.** Figures axis is invisible.

**Response:** The figure has been now changed into tabular form.

**Reviewer #2:**

**1.** The previous comments to the manuscript should have been incorporated in the resubmitted version. It was strongly suggested that the proof reading be done rigorously, but the current manuscript has many errors which could have been solved with proper proof reading. A few examples are as follows-
**Response:** Thank you for your feedback on our manuscript. We apologize for not incorporating previous comments and understand the need for rigorous proofreading. We have carefully addressed these issues in the revised version.

**2.** L20- “Genetically Male Tilapia” has already been mentioned to be referred to with “GMT”, yet L25, L27 mention “GMT (Tilapia)”

**Response:** The GMT (Tilapia) has been used in the entire manuscript**.**

**3.** L26- “Delicious” is not the proper adjective to use for amino acids, in a scientific research article.

**Response:** The name is now changed to Umami Taste in the revised manuscript

**4.** L28- Full stop/Period between “acid categories” and “with RMD”

**Response:** The sentence has been revised.

**5.** L45, L46- Use of “and” twice in the sentence.

**Response:** The sentence has been revised.

**6.** L58- Use of “IAAF” and then “IAAFS” for Integrated Agri-Aquaculture Farming SystemL58- “IAAFS” is not required to be in the bracket.

**Response:** The word IAAFS has been changed.

**7.** L60- Grammatical error- “improves” instead of “improve”

**Response:** The word improve has been replaced with improves.

**8.** L66-L68- The whole sentence needs restructuring to remove grammatical errors

**Response:** The sentence has been revised as, the co-culture system has been observed to reduce pest communities and lower the requirement for agrochemical inputs, including pesticides and herbicides.

**9.** L168- Both curly brackets and square brackets are used at once.

**Response:** The sentence has been revised.

**10.** L152- “Meat analyses” is very unclear, the specific tissues to be analyzed are to be mentioned.

**Response:** The meat analysis has been revised as given below, After the collection of fishes removed the scales, skin, and inter muscular thorn. Fish muscles were cut and merged into a sample; these samples were carefully packaged in labeled, airtight plastic bags and stored at -20°C for further analysis

**11.** The errors of the above-mentioned kind exist too frequently in the manuscript.

**Response:** We appreciated the feedback regarding the frequency of errors in the manuscript. We acknowledged the importance of addressing these issues to enhance the quality and credibility of our work. We thoroughly reviewed and revised the manuscript to rectify the mentioned errors and ensured its overall improvement.

**1.** The authors have stated that information on climate, elevation and temperature of the study area is essential to interpret the results of the study, yet there is no interpretation on that aspect in the discussion. There should also be a proper introduction for how these factors can be helpful in interpreting the results.

**Response:** The above information has been added to the introduction and discussion.

**2.** The experimental design states that, the study is conducted on 12 plots of 6500sq.ft each, fishes were stocked at a rate of 9000/ha, meaning approximately total 6500 fishes were stocked , of which only 3 fishes per plot were sampled for proximate and amino acid analysis? Were the fishes used in proximate analysis and Amino acid analysis the same? A sample size of three fish per plot is too small; the number of replicates should be more.

**Response:** we were stocked 9000/ha

Total sqft in one ha is 107693

Then calculate the total number of fish in a 6500sqft pond

=6500*9000/107693= 543.71 approximately 544

This means approximately 544 fish were stocked in a 6500sqft pond

12 experimental plots were used in this experiment.

Three fishes per plot means 36 samples for proximate and 36 fishes for an amino acid profile in this way a total of 72 samples were collected for analysis.

Proximate analysis: 3×12= 36

Amino Acid Profile: 3×12= 36

Total: 72

**3.** The experimental design is not clear.

**Response:** The experimental design has been clear in the revised manuscript

**4.** The authors have removed all information about zooplankton identification, but have responded to my previous query, hence I would like to mention that it is important to cite the taxonomic key and reference material used for zooplankton identification, as it not only helps the author to validate their work during the peer- review process, but also helps the reader to conduct similar study when the manuscript is published.

**Response:** The information has been added.
