# Supplementary material for: Optimizing rice-fish co-culture: Investigating the impact of rice spacing density on biochemical profiles and production of genetically modified tilapia (Oreochromis spp.) and Cyprinus carpio
Source: PLoS One. 2023 Dec 28;18(12):e0295996. doi: 10.1371/journal.pone.0295996 (PMC10754446; doi:10.1371/journal.pone.0295996)
Supplement: Attachment — Submitted filename: Point by point response.docx [file pone.0295996.s006.docx]

**RESPONSE LETTER**

The,

Editors,

PLOS ONE

**Subject: SUBMISSION OF REVISED PAPER**

Dear Editor,

Thank you for your email Dated: 15/Nov/2023, enclosing the reviewer’s comments. We appreciate the time and effort that you and the reviewers have dedicated to providing your valuable feedback on my manuscript. We are grateful to the reviewers for their insightful comments on my paper. We have carefully reviewed the comments and have revised the manuscript accordingly. Our responses are given in a point-by-point manner below. We hope the revised version is now suitable for publication and look forward to hearing from you in due course.

You’re sincerely,

**Muhammad Inayat**

**POINT-BY-POINT RESPONSE TO REVIEWER'S COMMENTS**

**Additional Editor Comments**

Dear authors

Your re-reviewed ms has come back with addition corrections. This is due to the fact that the previously two reviewers declined to take up your ms for further reviewing. As I see some potential in your ms, I have decided to give you another fair chance revise it further. Please note that your next revised ms (R4) will not be reivewed again. I will check it and send my observations to the Editor in Chief for his/her final decision. To increase the chances of positive consideration of your ms, please carefully revise your ms based on the observations of the two new reviewers. Plus please check the ms for grammatical, structural and/or typos.

**Response:** Thank you for providing the opportunity for further revision. We appreciate your consideration and the potential you see in our manuscript. We carefully revised the manuscript based on their feedback. Additionally, we conducted a thorough review to rectify any grammatical, structural, or typographical issues.

Please use the space provided to explain your answers to the questions above.

You may also include additional comments for the author, including concerns about dual publication, research ethics, or publication ethics. (Please upload your review as an attachment if it exceeds 20,000 characters)

**Reviewer #3:**

**1.** The manuscript has some new ideas and/or data.

**Response:** Thank you for recognizing the manuscript's novel ideas and/or data; we appreciate your positive feedback.

**2.** The authors are interested in rice production together with fish farming

**Response:** Thank you for your positive feedback.

3. The authors have considered the required variables of both these groups

**Response:** Thank you for your positive feedback.

4. Proximate composition of different elements has been considered.

**Response:** We appreciate your recognition of the comprehensive consideration of proximate composition for different elements in our study.

The manuscript has some weak aspects which may be addressed:
**1.** Discuss the role of temperature on the differential fatty acid composition.

**Response:**  The role of temperature on the differential fatty acid composition has been added.

**2.** The authors showed higher crude protein and fat content in genetically male tilapia tan *Cyprinus carpio*. Since fat composition is influenced by temperature, authors are advised to discuss this aspect.

**Response:** Thank you for the insightful suggestion, the above detail has been added.

**3.** Fish growth depends on plankton quality and quantity. Did the authors have any idea of these aspects in different treatments?

**Response:** In the rice-fish co-culture system, the author's detailed investigation of plankton quantity and quality under different treatments provided important insights into their potential effects on fish growth. The research highlights the significant role these factors play in determining the overall dynamics of the co-culture system as well as how different rice spacing densities affect phytoplankton and zooplankton ecosystems.

**4.** The fish species excrete considerable quantities of phosphorus and nitrogen compounds which influence the plankton dynamics.

**Response:** The research highlights the significant impact of fish species on the ecosystem by excreting significant quantities of phosphorus and nitrogen compounds. These excreted compounds play a pivotal role in affecting the dynamics of plankton within the system. By introducing additional nutrients, specifically phosphorus, and nitrogen, fish excretion becomes a key factor influencing the growth and composition of plankton communities. Understanding this aspect contributes to a more comprehensive understanding of the intricate relationships between fish, nutrients, and plankton dynamics in the studied ecosystem.

**5**. Did you measure the N:P ratios during the study period.

**Response**: No, I did not measure the N:P ratios during the study.

**Reviewer #4:** Optimizing Rice-Fish Co-Culture: Investigating the Impact of Rice Spacing density on Biochemical Profiles and Production of Genetically Modified Tilapia (*Oreochromis spp.*) and *Cyprinus carpio*

In general, the idea of the manuscript is of relevance due sustainability purposes. The evaluation of the impact of rice spacing density on the production of GMT and *Cyprinus carpio* is understood, however, the ideas in some sections are hard to understand. Authors may consider rewriting and revising again the editing and translation of certain concepts. Also, there are a lot of errors on spaces and endpoints. General trends in results are lacking. The discussion needs to be the discussion must be strengthened and conclusive, the importance of the general aim must be highlighted. Some of the references used in the introduction and discussion must be updated.

**Response:** We appreciate your feedback. We addressed clarity issues, revised editing and translation, corrected spacing and endpoint errors, included general trends in results, strengthened and concluded the discussion, and emphasized the importance of the general aim, Your input was valuable in refining the content.

Abstract.
GMT- specify

**Response:** The word GMT has been specified in the abstract

Line 73. Aquaculture

**Response:** The aquaculture word has been revised.
Line 80. Herbicides. Several

**Response:** The line has been revised.
Line 83. friendly. In

**Response:** The line has been revised.
Line 89-90. Please revise the writhing

**Response:** The line has been rewritten in the revised manuscript.
Line 93-95. The idea is not completely comprehensible

**Response:** This has been comprehensibly explained in the revised manuscript.

Line 96-98 unite ideas

**Response:** The ideas have been united in the revised manuscript.
Line 108. There are two points

**Response:** The line has been revised.
Line 111-112 authors may consider eliminating the idea, as the aim is established further in the document. Perhaps authors can consider adding the hypothesis of the study.

**Response:** These line has been updated.
Line 112 The study aims to evaluate the integration…

**Response:** The above information has been added.
Line 130. Eliminate propose. For the present assessment…

**Response:** The line has been revised.
Line 131-132 The idea is poorly understood

**Response:** These lines have been rewritten for better understanding.
Is there any reference used for the experimental design?
Line 146 study. The acclimatization…

**Response:** The line has been revised in the revised manuscript.
Line 148, 151, 157 double space

**Response:** Double space has been removed.
Line 167 the idea is vague, specifically the ending part of the idea.

**Response:** The idea has been updated.
References of the keys and manuals must be added to sample collection

Line 180 fish

**Response:** The line has been updated.

Line 180-Previous to the experiment

**Response:** The line has been revised.
Rewrite the idea
Is there any reference of the formula? Previous study were it has been used before?
Line 188 eliminate the

**Response:** The information has been revised.
The monitoring of water quality is vague. Which parameters?

**Response:** The water quality parameter has been added
Line 232. One way anova?

**Response:** The effects of different rice densities on the growth performance, meat proximate, and amino acid profile of two fish species were assessed through a factorial ANOVA using PROC GLM in SAS software (version 9.1). Both rice densities and fish species were treated as main effects, and their potential interactions were tested. This design allows for a comprehensive examination of the simultaneous influence of rice densities and fish species on the measured variables. Therefore, a factorial ANOVA was deemed appropriate for our study.
Line 233-235 rewrite the idea

**Response:** The idea has been re-written.
Line 278 are presented

**Response:** The line has been updated
Line 290 were presented?

**Response:** The line has been revised.
In the section of results, the general idea must be established and described. Line 298-299 is out of line.

**Response:** The idea has been established and line 298-299 has been improved.
Describe Plankton community trends
Line 311- according to what?

**Response:** The information has been added.
Line 326- according to what authors or compared to previous studies?

**Response:** The information has been added.
No trends of the results are considered in the discussion as well as the use of tables or figures.
Line 341-342 the authors may consider mentioning why…

**Response:** The information has been revised.
Line 359 elaborates idea
**Response:** The idea has been elaborated.

Fig 1 modify the map due to its poor quality

**Response:** The map is modified.
Fig 2 Experimental design considering 12 plots ()

**Response:** The information has been updated.

Fig 3. Standard deviation of how many replicates?

**Response:** In Fig 3. There are three replicates with each replicate
Table 1 and 2 describe the treatment groups in ()

**Response:** The information has been revised.
